# Supplementary material for: Phosphorylation regulates the binding of intrinsically disordered proteins via a flexible conformation selection mechanism
Source: Commun Chem. 2020 Sep 7;3:123. doi: 10.1038/s42004-020-00370-5 (PMC9814494; doi:10.1038/s42004-020-00370-5)
Supplement: Supplementary file 1 — Supplementary Information [file 42004_2020_370_MOESM1_ESM.pdf]

Supporting Information

Phosphorylation regulates the binding of intrinsically disordered proteins via a flexible conformation selection mechanism

Na Liu[1,2†], Yue Guo[1,3†], Shangbo Ning[1,2], Mojie Duan[1*]

1. *Key Laboratory of magnetic Resonance in Biological Systems, State Key Laboratory of Magnetic Resonance and Atomic and Molecular Physics, National Center for Magnetic Resonance in Wuhan, Wuhan Institute of Physics and Mathematics, Chinese Academy of Sciences, Wuhan 430071, People's Republic of China*

2. *School of biological and pharmaceutical engineering, Wuhan Polytechnic University, Wuhan 430023, People's Republic of China*

3. *University of Chinese Academy of Sciences, Beijing 100049, People's Republic of China*

†: These authors contribute equally to this work.

* **Corresponding author:**
Mojie Duan
Email: mjduan@wipm.ac.cn
Tel: +86-27-87197173

Supplementary Methods

Simulation details of PTMetaD-WTE and BE-MetaD. The system would escape the energy minima quickly by accumulating history-dependent Gaussian potential (bias potential) on specific collective variables (CVs) in the metadynamics simulations.[1] By combining with the parallel tempering (PT) and replica exchange schemes, many advanced metadynamics technologies were developed, such as PTMetaD-WTE and BE-MetaD. These methods could achieve higher efficiency and consider multiple collective variables in the simulations, therefore, could be used in the study of large biological systems. [2-4]

In this work, PTMetaD-WTE was employed to investigate the structure ensemble of isolated pKID and KID. Two CVs are selected: *α-score* for residues 120-129 and *α-score* for residues 134-144. These two CVs are used to measure the number of 6-residue segments that form α-helix, the calculation of this CV as follow:

$$\alpha - score = \sum_i \frac{1-\left(\frac{r_i}{0.08}\right)^8}{1-\left(\frac{r_i}{0.08}\right)^{12}}$$

where the bias factor γ was set to be 16, the height of initial bias is 1.0kJ/mol and widths of 0.2 rad, MetaD bias was deposited every 500 steps, where each step 1.5fs. Exchange of configurations between neighboring replicas was attempted every 750 fs.

Bias-exchange Metadynamics (BE-MetaD) simulation was employed for the study of pKID/KID and KIX binding. The metadynamics bias was applied to four CVs: *α-score* for residues 120-129 on pKID or KID (CV1), *α-score* for residues 134-144 on pKID or KID (CV2), the COM distance between pKID/KID and KIX (CV3), the number of native contacts between pKID/KID and KIX (CV4). The α-score CVs were employed to describe the folding of pKID, the CV3 was used to depict the binding process and the CV4 describes the progress of binding between pKID/KID with KIX. The COM distance in CV3 was limited less than 4.0 nm with harmonic restrained potential during the simulation to focus sampling on the relevant regions of configurational space. The harmonic potential with the following form:

$$V_M = \begin{cases} \frac{1}{2}k(S-S_0)^2, & if\ S > S_0 \\ 0, & if\ S \le S_0, \end{cases}$$

where *S* corresponds to the COM distance between pKID/KID and KIX. S_0 is 3.0 nm. k is the force constant 500 kJ/(mol $\cdot\, nm^2$). The CV4 was calculated as a sum of switching function:

$$Q = \sum_{ij} \frac{1}{1+\exp(\beta(r_{ij}-\lambda r_{ij}^0))}$$

where r_{ij} represents the com distance between heavy atoms in pKID/KID and KIX whose com distance are closer than 0.45 nm in the experimetal structure. And r_{ij}^0=0.45,

λ=1.8, β=50 nm^{-1}.

In the BE-MetaD simulation, Gaussian potential height *w* was adopted 2.0 kJ/mol for all CVs, Gaussian width was 0.2 for CV1 to CV3 and 10 for CV4. The bias factor is set to 32 in all replicas, the Gaussian bias was deposited every 5 ps.

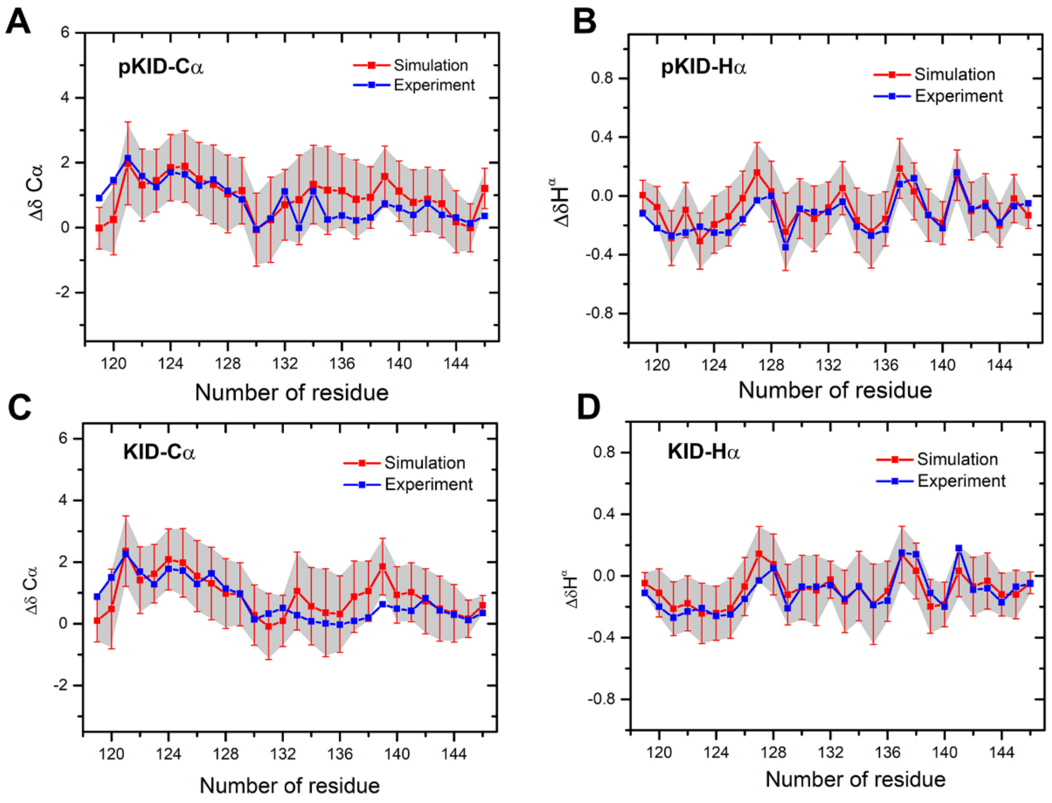

Supplementary Figure 1. Comparison of computational predicted and experimental measured secondary chemical shifts of free pKID and KID. (A) Secondary chemical shifts of Cα atoms of free pKID; (B) Secondary chemical shifts of Hα atoms of free pKID; (C) Secondary chemical shifts of Cα atoms of free KID; (D) Secondary chemical shifts of Hα atoms of free KID. The standard deviations of computational values were given in the red shadows.

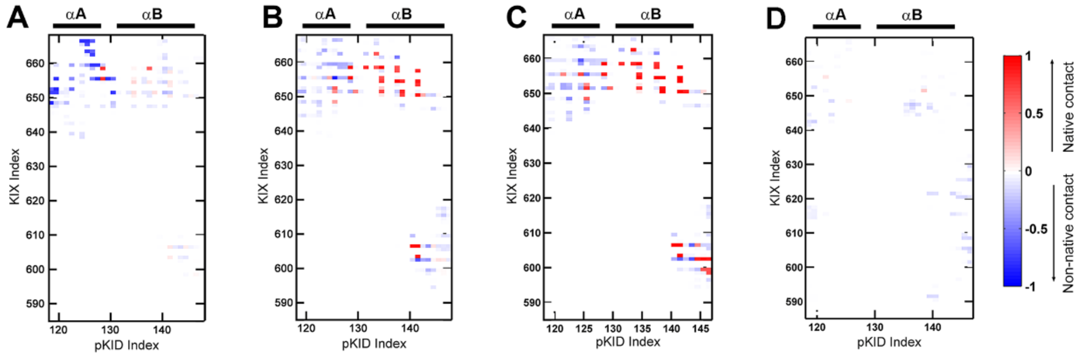

Supplementary Figure 2. Contact maps between residues on pKID and KIX of different states in the binding process. (A) In the hidden state H; (B) In the transition state T; (C) In the fully bounded state B. (D) In the free state F.

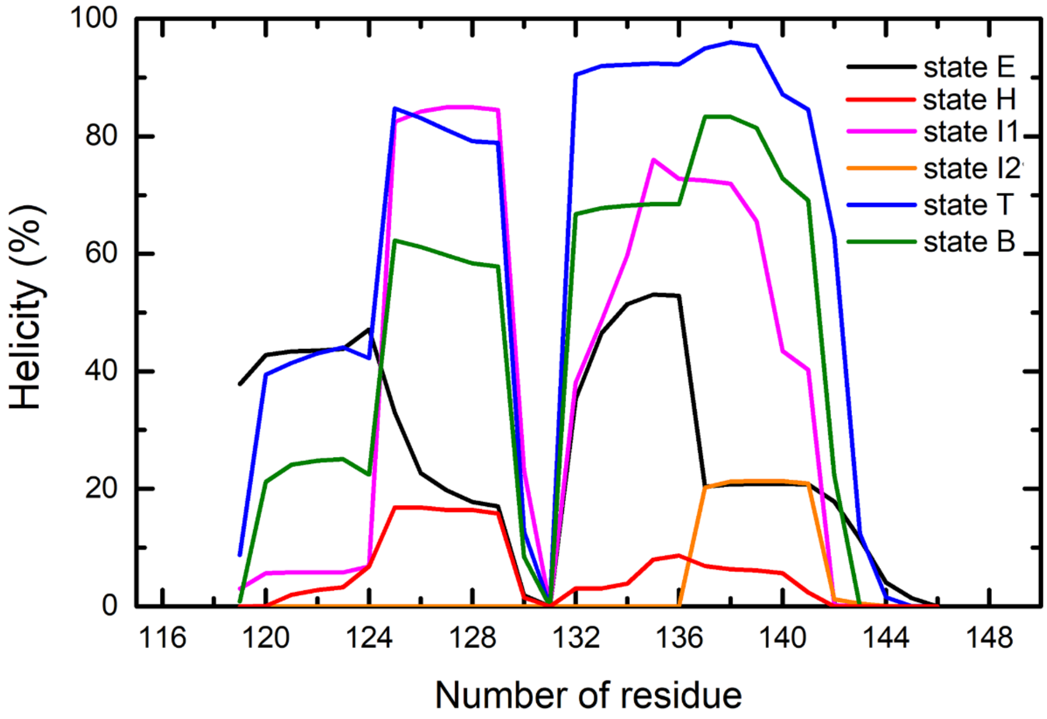

Supplementary Figure 3. The residue helicity of pKID for different free energy minima in the binding process of pKID and KIX.

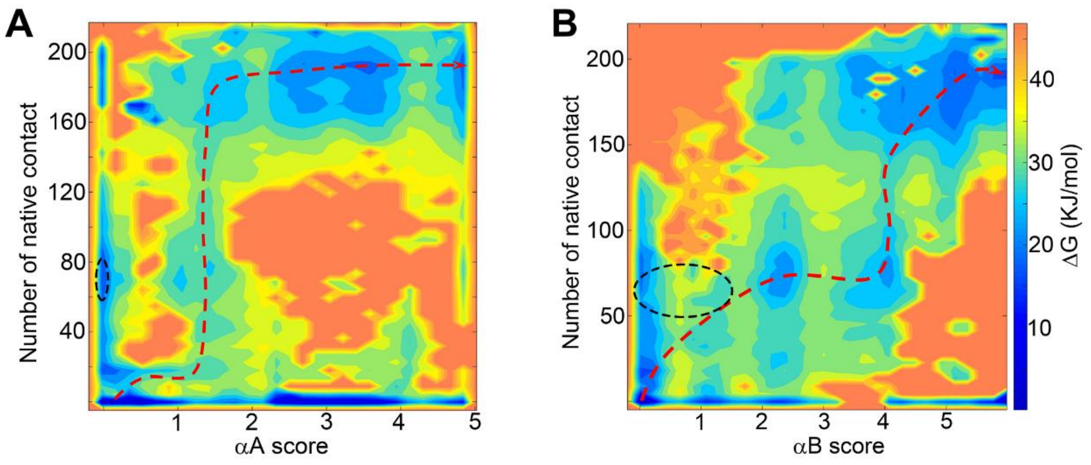

Supplementary Figure 4. The binding and folding process of pKID. (A) The free energy surfaces as the functions of αA helicity and the number of native contacts. (B) The free energy surfaces as the functions of αB helicity and the number of native contacts. The conformations in the intermediate I2 were labeled by the black dashed circles on the free energy surfaces.

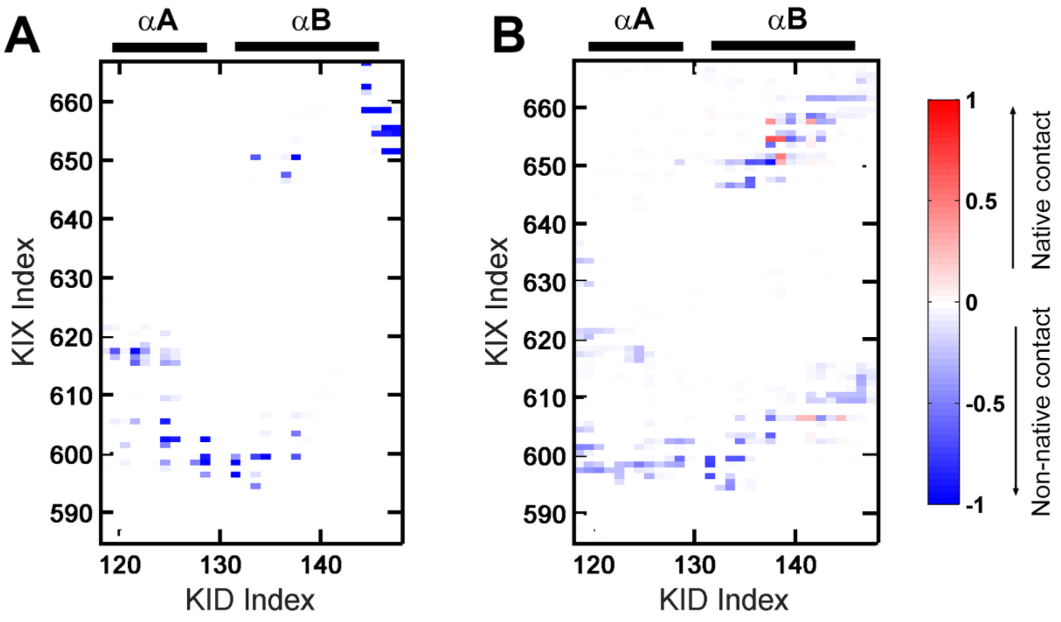

Supplementary Figure 5. The residue contact maps between KID and KIX. (A) In the encounter complex E; (B) In the intermediate state I.

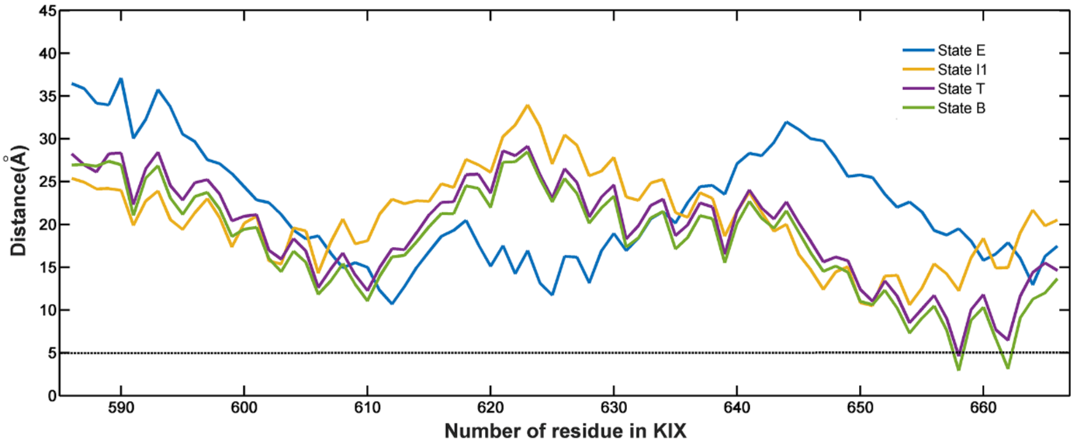

Supplementary Figure 6. The minimum distances between pSer and residues in KIX in different states of pKID-KIX binding.

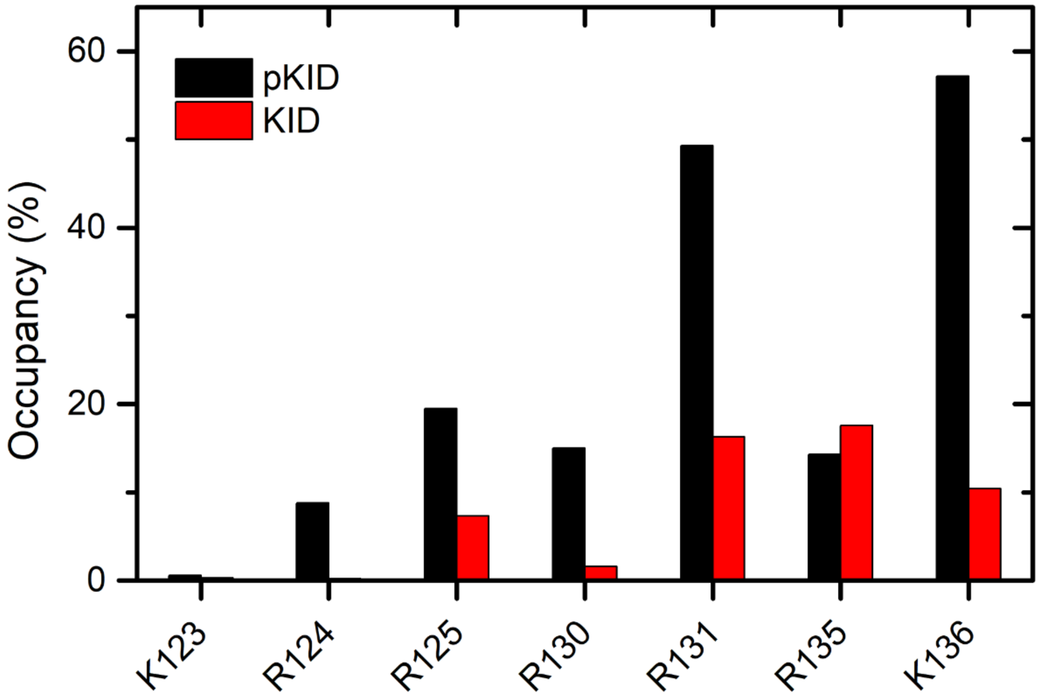

Supplementary Figure 7. Side-chain contact probability between residue 133 and the positive charged residues on free pKID and KID.

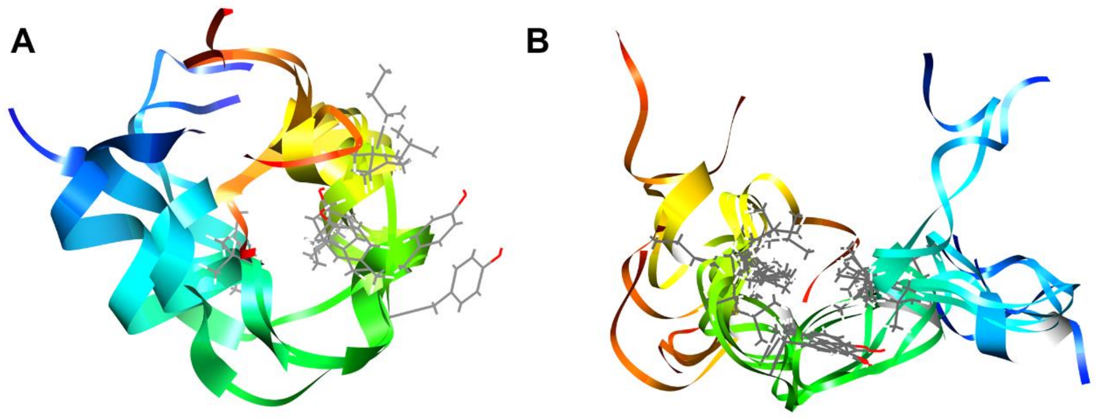

Supplementary Figure 8. The representative structures with hydrophobic residue cluster (HRC) in free pKID and the hidden state of pKID-KIX binding process. (A) In free pKID. (B) In the hidden state (H). The hydrophobic residues (Leu128, Tyr134, Leu137, Leu138) are represented by wires.

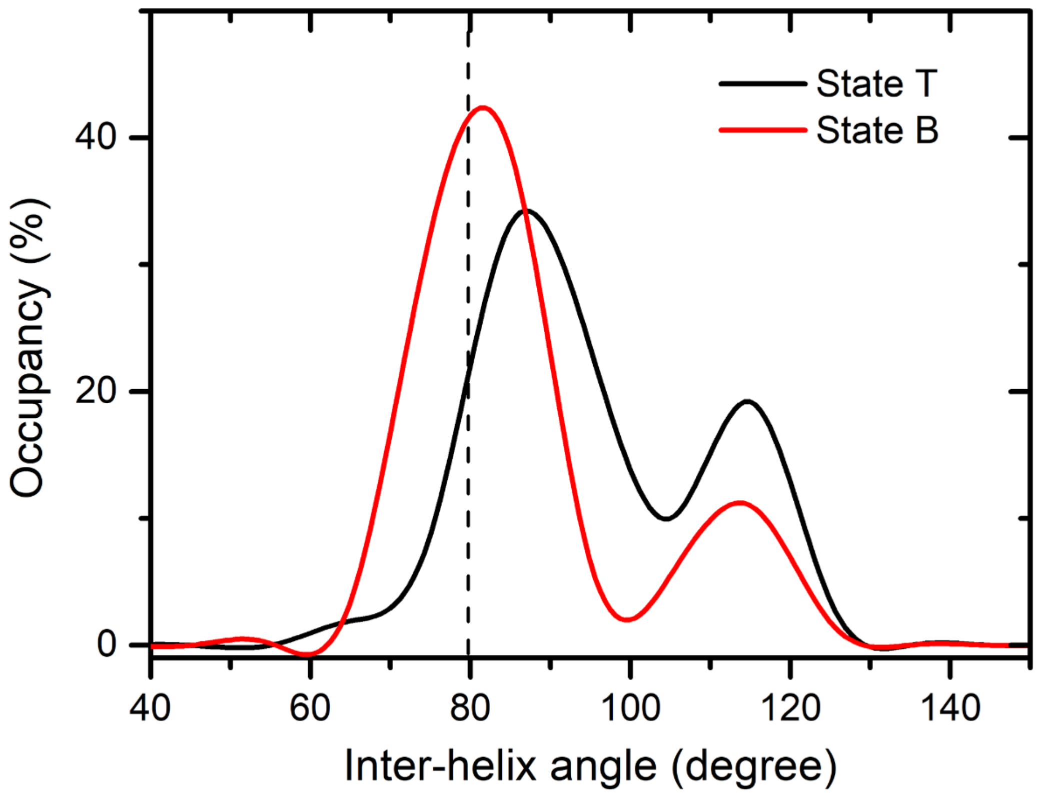

Supplementary Figure 9. The distributions of αA-αB angles in different states of pKID-KIX binding process. The αA-αB angle is 79.8^{o} in the experimental complex structure (PDB ID:1KDX), which is labeled by the dashed line.

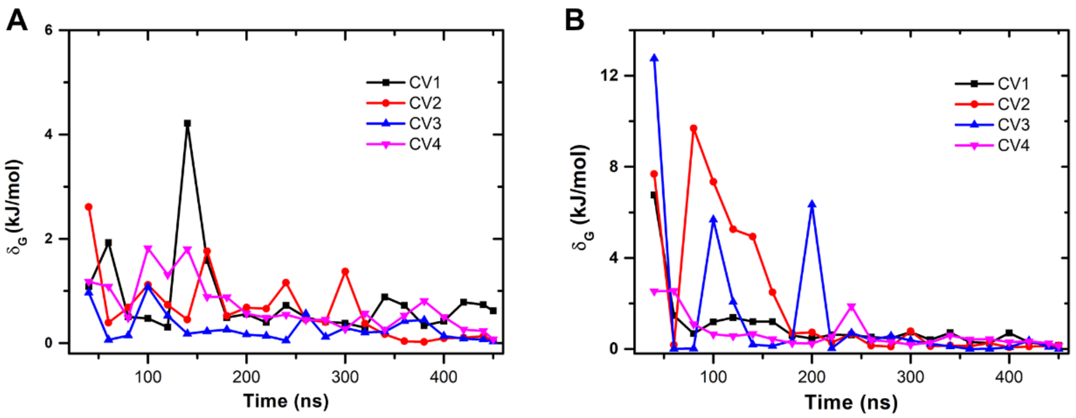

Supplementary Figure 10. The convergence tests of BE-MetaD simulations. (A) pKID-KIX binding simulation; (B) KID-KIX binding simulation. The convergence of BE-MetaD simulation was estimated by the free energy profile changes as a function of simulation time. δ_G is corresponding to the free energy change from time $t - \delta t$ to t, where δt=30 ns. The simulations are basically converged after 200 ns.

Supplementary Table 1. Helicity of free pKID and KID measured by experiments and predicted by simulations.

Region		Experiment	This work
pKID	α_A	50-60%	45.6%
	α_B	~15%	18.9%
KID	α_A	50-60%	51%
	α_B	~10%	14.6%

Supplementary References:

1. Laio A, Gervasio FL. Metadynamics: a method to simulate rare events and reconstruct the free energy in biophysics, chemistry and material science. Reports on Progress in Physics 2008, **71**(12).
2. Barducci A, Bonomi M, Parrinello M. Metadynamics. Wiley Interdisciplinary Reviews-Computational Molecular Science 2011, **1**(5)**:** 826-843.
3. Barducci A, Bussi G, Parrinello M. Well-tempered metadynamics: a smoothly converging and tunable free-energy method. Physical Review Letters 2008, **100**(2)**:** 020603.
4. Piana S, Laio A. A bias-exchange approach to protein folding. J Phys Chem B 2007, **111**(17)**:** 4553-4559.
