# Supplementary material for: Phosphorylation regulates the binding of intrinsically disordered proteins via a flexible conformation selection mechanism
Source: Commun Chem. 2020 Sep 7;3:123. doi: 10.1038/s42004-020-00370-5 (PMC9814494; doi:10.1038/s42004-020-00370-5)
Supplement: Supplementary file 2 — Description of Additional Supplementary Files [file 42004_2020_370_MOESM2_ESM.pdf]

**Description of Additional Supplementary Files**

File Name: Supplementary Data 1
Description: The coordinates of representative structures of the minima on the free energy surface of pKID-KIX binding process

File Name: Supplementary Data 2
Description: KID-KIX binding process
